# Temperature sensitivity and temperature response across development in the *Drosophila* larva

**DOI:** 10.3389/fnmol.2023.1275469

**Published:** 2023-10-27

**Authors:** Anastasiia Evans, Anggie J. Ferrer, Erica Fradkov, Joseph W. Shomar, Josh Forer, Mason Klein

**Affiliations:** ^1^Department of Physics, University of Miami, Coral Gables, FL, United States; ^2^Department of Biology, University of Miami, Coral Gables, FL, United States

**Keywords:** thermotaxis, navigation, *Drosophila*, development, neurophysiology, sensitivity

## Abstract

The surrounding thermal environment is highly important for the survival and fitness of animals, and as a result most exhibit behavioral and neural responses to temperature changes. We study signals generated by thermosensory neurons in *Drosophila* larvae and also the physical and sensory effects of temperature variation on locomotion and navigation. In particular we characterize how sensory neuronal and behavioral responses to temperature variation both change across the development of the larva. Looking at a wide range of non-nociceptive isotropic thermal environments, we characterize the dependence of speed, turning rate, and other behavioral components on temperature, distinguishing the physical effects of temperature from behavior changes based on sensory processing. We also characterize the strategies larvae use to modulate individual behavioral components to produce directed navigation along thermal gradients, and how these strategies change during physical development. Simulations based on modified random walks show where thermotaxis in each developmental stage fits into the larger context of possible navigation strategies. We also investigate cool sensing neurons in the larva's dorsal organ ganglion, characterizing neural response to sine-wave modulation of temperature while performing single-cell-resolution 3D imaging. We determine the sensitivity of these neurons, which produce signals in response to extremely small temperature changes. Combining thermotaxis results with neurophysiology data, we observe, across development, sensitivity to temperature change as low as a few thousandths of a °C per second, or a few hundredths of a °C in absolute temperature change.

## 1. Introduction

Temperature is nearly universally important in biology, due to its influence on chemical reaction rates, and it strongly affects systems across a vast range of length and time scales (Arroyo et al., 2022). For organisms, the surrounding temperature in isotropic conditions helps determine the state of internal processes and external physical actions. For motile organisms in particular, response to temperature variation in the surrounding environment is crucial for their ability to survive and reproduce (Sengupta and Garrity, 2013), and thermotaxis (movement along temperature gradients) can be seen in even the simplest creatures (Paulick et al., 2017; Zhong et al., 2022). Because temperature is a simple scalar quantity that is readily controllable in a laboratory setting with reasonably high precision, because it is so important for the fitness of organisms, and because of its considerable spatial and temporal variation in natural settings, it is desirable to reach a comprehensive understanding of how temperature impacts living systems.

From a neuroscience perspective, we would want to investigate how a stimulus input like temperature is converted into physical output like locomotion, and specifically to understand this process at the molecular, cellular, and behavioral levels. For example: How are neurons able to sense temperature? What signals do they send downstream and how are such signals processed? What are the behavioral actions and strategies deployed to reach improved thermal conditions? And finally, how do the answers to these questions change as the organism develops? A complete, step-by-step map of this entire process has not been achieved for any stimulus in any animal, due to the scale and complexity of most nervous systems.

Moving toward a more comprehensive understanding of salient behaviors like temperature response is more tractable in simpler model systems like the fruit fly *Drosophila melanogaster* (Roberts, 2006), and the even simpler larval *Drosophila* used in our work here. The fly larva responds robustly to both heating and cooling (Luo et al., 2010). It has a small number of neurons, is optically transparent and thus well-suited for *in vivo* imaging, and because it is a fly there are abundant genetic tools (Duffy, 2002; Jenett et al., 2012) for investigating molecular and cellular mechanisms that underlie behavior. The larva is a slow-moving animal with a limited, quantifiable behavioral repertoire, which allows for precise behavioral classification and higher throughput experiments. Larva development is also well defined and larvae grow very quickly, with only around four days between eclosion and pupariation. For these reasons, we utilize this model system in our present study of how thermal response in sensory neurons and behavior (exploration and navigation) varies across development.

**Figure 1:**
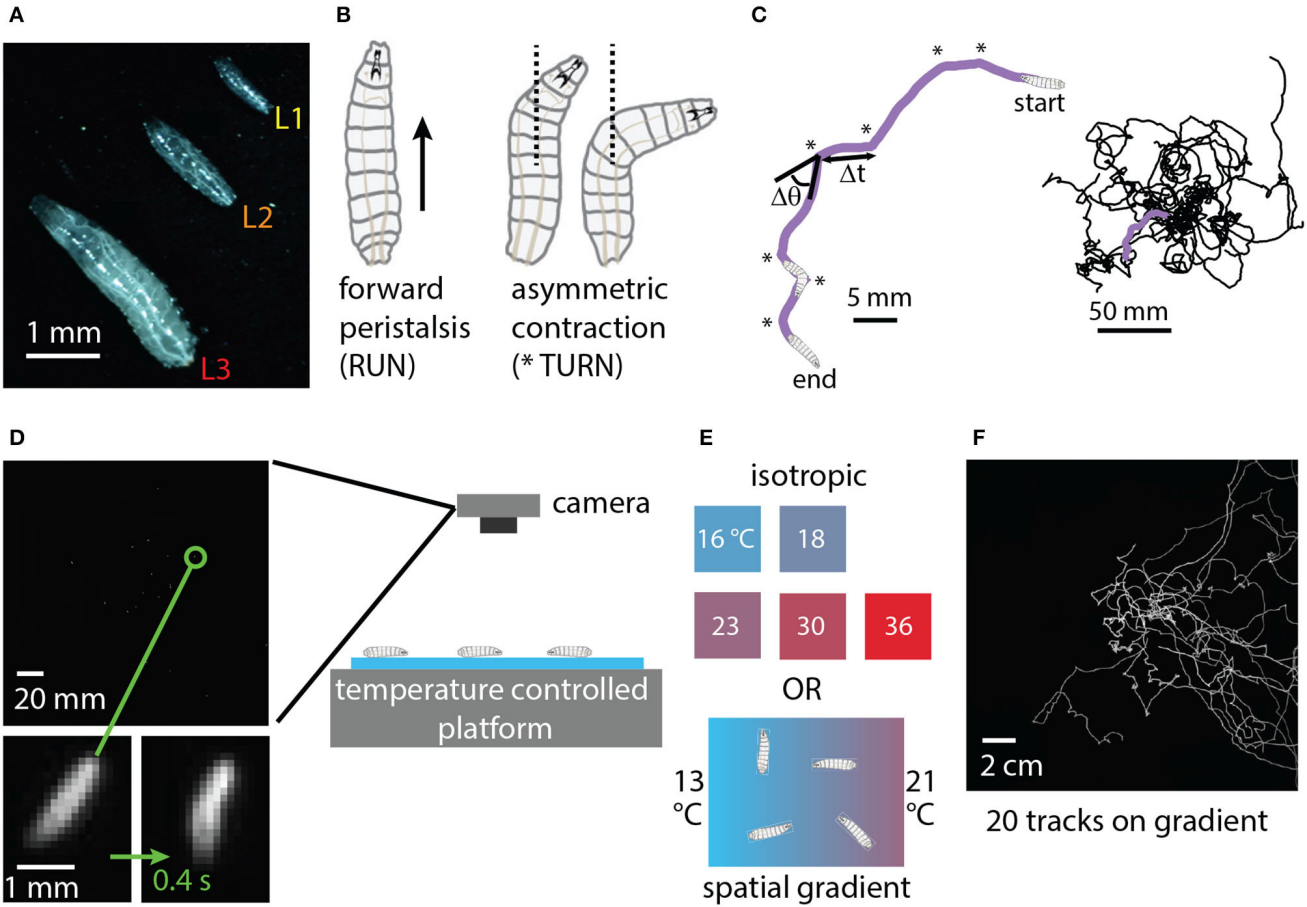
Experimental design: crawling larva in isotropic and thermal gradient environments. **(A)** Photograph of three adjacent *Drosophila* larvae crawling on black agar, one of each instar developmental stage (L1, L2, L3). **(B)** Schematic of the two primary physical actions taken by larvae crawling on a flat surface in the absence of food. Peristaltic waves of muscle contraction carry larvae forward (RUN), and asymmetric contractions alter the crawling direction (TURN) before forward motion resumes. **(C)** Sequences of runs and turns lead to random-walk-style trajectories. Left: purple path of a second instar larva (L2) crawling for 5 min. Turns are indicated by * symbols. Δ*t* indicates the duration of a run, and Δθ the direction change for the turn that proceeds the run. Right: trajectories for 20 larvae crawling together. **(D)** A CCD camera captures an image of a group of 20 larvae crawling on agar gel in the behavior arena, with a single zoomed-in larva shown at the beginning and end of a 0.4 s turn action. **(E)** Temperature control platforms produce either constant-temperature conditions (16, 18, 23, 30, or 36°C) or a spatial linear gradient in the *x*-direction of strength 0.4°C/cm (13°C on the cold side and 21°C on the warm side). **(F)** Full trajectories for 20 s instar larvae performing directed thermal navigation on the spatial linear gradient. The image is the sum of all frames of a 15 min video acquired by the camera.

The physical characteristics and locomotion of the *Drosophila* larva are shown in Figure 1. Larvae grow rapidly through three instars (denoted L1, L2, L3), with molting in between. Confined to a flat surface without food present, their motion can be described almost entirely in terms of two simple motor programs: forward crawling via peristalsis (“runs”) and asymmetric muscle contraction proceeded by more forward crawling (“turns”) (Lahiri et al., 2011). Such motion is akin to a random walk (Berg, 1993; Codling et al., 2008) with trajectories built from an alternating sequence of runs and turns (Figure 1C). The dominant behavioral features of exploratory locomotion are the crawling speed *v*, average time between turns Δ*t* (or equivalently the average turning rate *R* = 1/Δ*t*), the size of turns Δθ and the turn direction (whether Δθ is positive or negative).

Exploration and navigation have been studied in *Drosophila* larvae responding to nociceptive (Tracey et al., 2003; Turner et al., 2018) and non-nociceptive temperature (Rosenzweig et al., 2008; Luo et al., 2010; Klein et al., 2015), along with many other stimuli such as light intensity (Xiang et al., 2010; Keene and Sprecher, 2012; Kane et al., 2013), chemical concentration (Kreher et al., 2008; Gershow et al., 2012), mechanical vibration (Ohyama et al., 2013; Berne et al., 2021), sound (Zhang et al., 2013), etc. Larva perform navigation using the parameters (*v*, *R*, Δθ) noted above, but modulate them based on environmental conditions (Gomez-Marin and Louis, 2012), and in the case of spatial stimulus gradients, on their crawling direction. Biasing these parameters, for example by turning more often when crawling toward the cold side of a thermal gradient, leads to directed navigation alongside diffusive exploratory locomotion (Klein et al., 2017). Importantly, there have been characterizations of thermotaxis in L1 (Luo et al., 2010), L2 (Klein et al., 2015), or L3 (Sokabe et al., 2016) larvae, but to our knowledge there has not been a systematic characterization of larva behavioral response across the developmental stages.

**Figure 2:**
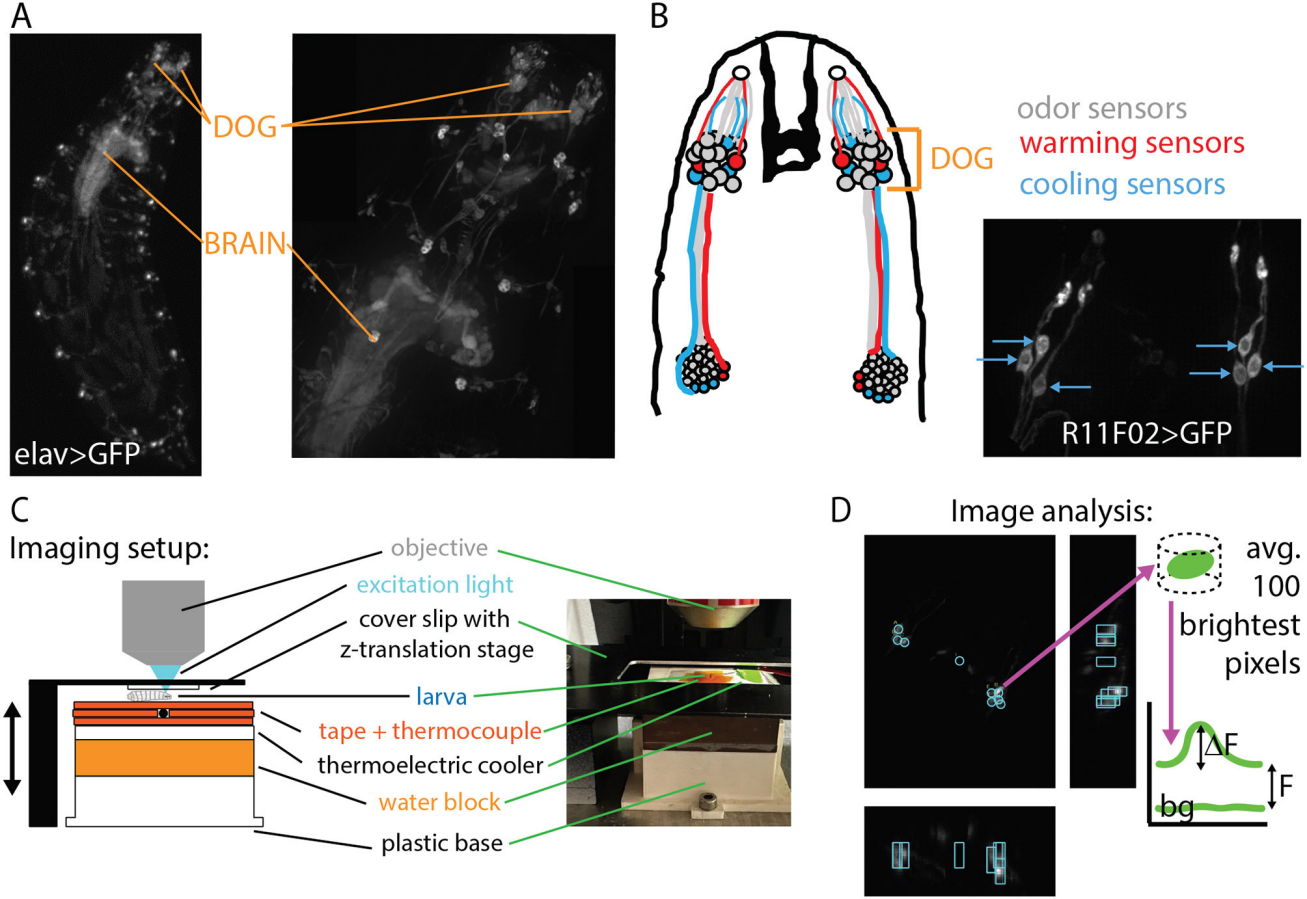
Experimental design: *in vivo* neurophysiology during controlled temperature modulation. **(A)** Nervous system of the *Drosophila* larva visualized with GFP under control of the elav pan-neuronal driver. Maximum intensity projections (MIPs) of 3D images acquired with a spinning-disk confocal microscope at 4× magnification (left) and 20× magnification (right). The dorsal organ ganglion (DOG) and central brain regions are indicated with orange lines. **(B)** Left: schematic of the thermosensory neurons of interest. Each DOG houses over 30 olfactory receptor neurons, along with three cooling sensors and two warming sensors. Right: MIP of the cooling sensor neurons labeled with the R11F02 driver. **(C)** Schematic and photograph of the temperature control system housed beneath the microscope objective. The larva is secured by gentle compression from a cover slip connected to a *z*-translation stage. Temperature is measured by a thermocouple probe beneath the larva and controlled by a thermoelectric cooler (TEC). **(D)** Neuron signal extraction. Cooling sensor neurons express the optical calcium indicator GCaMP6m. MIPs in the *z*- (larger panel), *x*-, and *y*-directions are used to locate neurons, then the brightness is determined with analysis software, and the Δ*F*/*F* vs. time signal is determined using baseline and background levels.

The molecular mechanisms underlying temperature sensation in general (Jordt et al., 2003; Dhaka et al., 2006) and in adult and larval *Drosophila* (Gallio et al., 2011; Ni et al., 2016; Budelli et al., 2019; Omelchenko et al., 2022), have been studied and characterized. The cellular mechanisms are partly understood, with cool sensing cells identified in the dorsal organ ganglion (DOG) (Klein et al., 2015) and possibly the terminal organ ganglion (TOG) (Liu et al., 2003), and warm sensing cells in the DOG (Hernandez-Nunez et al., 2021) (see Figure 2). There has also been a recent treatment of how the warming and cooling sections of the neural circuit combine to produce behavior (Hernandez-Nunez et al., 2021), but a complete circuit-level understanding of thermotaxis has not yet been achieved. Partially due to the difficulty of imaging larger larvae *in vivo*, a characterization of neuronal activity in response to temperature has not been performed across developmental stages.

In this paper, we focus on non-nociceptive cold avoidance behavior in the *Drosophila* larva, along with the responses of the primary cool sensing neurons in the DOG. We seek to understand the animal's response to temperature across development, especially its sensitivity to small temperature changes. We similarly seek to characterize neural response across development and determine the sensitivity of the sensory neurons to small temperature changes. We accomplish this by recording and analyzing groups of crawling larvae in isotropic and thermal gradient arenas, and by 3D confocal calcium imaging of neurons during controlled temperature modulation. Comparing results from both sensory neurons and navigation should illuminate connections between cellular and behavioral components of the organism's overall response to temperature.

## 2. Materials and methods

### 2.1. Fly strains and larva preparation

The strains used in this paper include wild-type Canton-S, R11F02>GCaMP6m for imaging, Ir25a^2^ mutants, a heterozygous Ir25a>TeTxLC (tetanus toxin light chain expressed in cells with IR25a), and an Ir25a rescue, which is Ir25a>Ir25a in an IR25a mutant background. Flies were housed and raised in tubes with cornmeal and molasses food at room temperature. The larvae used for imaging and behavior experiments were raised in cages with plates of an agar and grape juice mixture, plus inactive yeast added to the top of the gel. Grape juice plates were swapped from cages every 24 h, and larvae were selected from older plates. For staging specific instars, first instars (L1) were taken from plates 24–48 h after egg laying (AEL); second instars (L2) were taken from plates 48–72 h AEL; early third instars (L3 or L3a) 72–96 h AEL; mid third instars (L3b) 96–120 h AEL; late third instars (L3c) 120–144 h AEL. In addition to using AEL as a reference, each individual larva was screened manually for size and spiracle development. After selection from plates, larvae were rinsed in DI water and allowed to crawl in a plain agar transfer plate for several minutes before starting a behavior or imaging experiment.

### 2.2. Behavior: crawling arenas

Two types of temperature-controlled arenas were used for larva behavior experiments (Figure 1). The thermotaxis arena (previously used in Klein et al., 2015, 2017) established a linear gradient between 13 and 21°C on the agar gel (22 × 22 cm) in the horizontal (*x*) direction. Stable temperatures were maintained by two symmetrically arranged thermoelectric coolers (TECs) on the cold side, and by resistive cartridge heaters on the warm side, both regulated by PID controllers. The resulting spatial gradient of ~0.4°C/cm was checked before and after each experiment.

In the isotropic arena, temperature was controlled by four symmetrically arranged TECs underneath a 2.5 cm thick aluminum plate. Each TEC was secured between the aluminum plate and a copper water block, with chilled water circulating through all four water blocks in series to dissipate excess heat. Temperature was maintained with a PID feedback loop using a thermocouple probe secured to the top of the agar surface. A moat was built around the outside, with the edge of the gel in contact, and the moat water was replenished continuously to prevent gel desiccation (important especially at higher temperatures). Spatially uniform temperatures were achieved, and the values used were either 16, 18, 23, 30, or 36°C. We note that the entire range of temperatures used in all our experiments avoids both cold (Turner et al., 2018) and heat (Tracey et al., 2003) nociceptive regimes.

For both isotropic and spatial gradient arenas, around 20 larvae were placed near the center of an agar gel (2.5% wt./vol. agar, 0.75% wt./vol. charcoal) for each experiment, and were recorded for 15 min. Both arenas were enclosed in a chamber that blocks outside light.

### 2.3. Behavior: data acquisition

A 5-megapixel camera with an 8 mm lens was placed above the crawling arena, and the camera recorded larvae crawling from overhead at 15 frames per second. Larvae were visible due to dark field illumination from red LED strips arranged in a square, pointing inward along the edges of the arena. Because *Drosophila* larvae do not see this color, their behavioral response should be based only on the provided thermal stimulus. Raw images were processed using the MAGAT Analyzer system (Gershow et al., 2012), where images are converted into tracks consisting of the position and contour of every larva at every frame of the movie. Tracks are segmented into runs and reorientations (turns). All analysis after this step was performed using customized programs from either Matlab or Igor Pro.

### 2.4. Behavior: data analysis

Larva tracks were filtered out if they were excessively slow, or too short. Tracks end due to (1) the experiment ending, (2) leaving the field of view of the camera, but can also end when (3) larvae collide and identity cannot be maintained or (4) the camera loses track of the larvae. To ensure that one larva corresponds to only one track, we also remove tracks that are shorter than 300 s in duration or start more than 300 s after the beginning of an experiment.

Customized analysis software in Matlab produces a table of information about each run or each track, and determines: Δ*t*, the duration of the run; the initial (*x*_0_,*y*_0_) and final (*x*_1_,*y*_1_) positions of the run; θ, the average crawling direction of the run (0° indicates the +*x* direction, 90° the +*y* direction, etc.); whether the run ended in a turn (each run has *n* turns), and if there is a turn, the larva's orientation before (θ_1_) and after (θ_2_); and ℓ, the path length of the run.

Using these quantities we then used customized software written in Igor Pro to calculate behavioral parameters larvae typically use to modulate navigation and exploration: the turning rate *R* = Σ*n*/ΣΔ*t*; the crawling speed during runs *v* = Σℓ/ΣΔ*t*; the angle of each turn Δθ = θ_2_ − θ_1_; the efficiency of each run $\ell /\sqrt{{\left(x_{1}-x_{0}\right)}^{2}+{\left(y_{1}-y_{0}\right)}^{2}}$, and the temperature change experienced by the larva during a run *dT*/*dt* = *v* × cosθ × Δ*T*/Δ*x*, where Δ*T*/Δ*x* is the spatial thermal gradient steepness.

When analyzing thermotaxis experiments, the same quantities can be computed, but for subsets of runs grouped by crawling direction. This could determine, for example, that turning rate *R* is higher when larvae crawl toward the cold side and lower when they crawl toward the warm side.

A summary number of navigation efficiency, the navigation index, is also computed for each condition. The navigation index for each larva is the average *x*-component of its velocity during runs, divided by its average speed during runs: *NI* = 〈*v*_*x*_〉/〈*v*〉. This value is computed separately for each individual larvae, and then the navigation index reported for different conditions is the average of *NI* for all larvae in the experiment set.

Sensitivity to temperature change was calculated by grouping all runs into subsets based on the average *dT*/*dt* of each run. We then compared turning rates under different *dT*/*dt* conditions, then used two methods to determine statistical significance. First, we extracted a list of all run durations Δ*t* for each condition and computed the *p*-value using Student's *t*-test. If the turning rate comparison yielded *p* < 0.05 then we considered the behavioral difference statistically significant and interpret this as larvae being sensitive to temperature changes by at least the difference between *dT*/*dt* values in the two groups. Uncertainty for each turning was determined by dividing the variance by $\sqrt{n}$, where *n* is the number of turns. We assumed Poisson statistics, where the mean and variance are equal (*n*/*T*, with *T* the total time not spent turning).

Second, as a more conservative and realistic method for determining uncertainty that does not assume a purely Poisson distribution of times between turns, we deployed a bootstrapping approach. For data drawing from *N*_*exp*_ experiments, we randomly sample (with replacement) *N*_*exp*_ times, and for each experiment we sample, we randomly sample tracks (with replacement) *N*_*tracks*_ times (*N*_*tracks*_ is the number of tracks for the specific experiment), and from there randomly sample (with replacement) turns *n* times and extract the time duration Δ*t* of the preceding run (*n* is the number of turns whose preceding run was in the appropriate crawling direction or *dT*/*dt* range). The whole sampled set of Δ*t* are averaged, and the inverse is the turning rate *R*. The above sampling method is repeated 1000 times, and the standard deviation of set of 1,000 *R* values reflects the uncertainty in *R*. Significance is then determined by examining the list of 1,000 averaged *R* values for two different conditions (say, two cooling rates). For conditions 1 and 2, if *R*_1_ > *R*_2_ 95% of the time, then *p* < 0.05 for the claim that *R*_1_ > *R*_2_.

The second approach yielded identical means, but higher uncertainty in all our measured *R* values, and we use this method for the error bars appearing on any *R* plot, and use its criterion for statistical significance.

### 2.5. Neurophysiology: image acquisition

Larva of the strain R11F02>GCaMP6m (homozygous expression of the calcium indicator GCaMP6m in the primary cold sensor neurons, Figure 2A) were imaged with an upright spinning disk confocal microscope (Andor Dragonfly 200), which illuminates the larva with blue laser light (488 nm) and detects emitted green light with an EMCCD camera. The stage under the microscope moved vertically after each image slice, producing a 3D image, and the process was repeated for several minutes. Imaging was performed with either 20× or 40× magnification. Both are air objectives, which was important in preventing the objective from acting as a heat sink during thermal control.

### 2.6. Neurophysiology: temperature control and larva immobilization

We controlled the temperature of the larva during neurophysiology video recording with a custom-built stage beneath the microscope objective (Figure 2C). Temperature was measured with a thermocouple probe (precision 0.1°C) secured to one side of a TEC with thermally conductive (and electrically insulating) polymide tape, and larvae were placed just above the probe. Temperature was set with a PID controller, and the set value could be changed over time using customized software written in LabVIEW (also used in Klein et al., 2015), in a sine wave pattern. The amplitude was between 0.05 and 1.0°C (constant for each recording), the period was 60 s, and the offset level was 17°C, matching the starting conditions of larvae on the thermal gradient arena. Each larva was imaged for 30 s before sine wave modulation started, and at least two periods of modulation were recorded.

Larvae were held in place using gentle compression from a cover slip attached to a 1.5 mm thick aluminum frame, itself secured to a vertical translation stage. The cover slip was lowered from above until the animal ceased moving.

### 2.7. Neurophysiology: image analysis

After recording 3D movies of neuron fluorescence, we used customized software written in Igor Pro to extract signal levels (Figure 2D). The user draws a pillbox shape surrounding each neuron or region of interest, and adjusts the position of pillboxes for subsequent time points of the movie (3D image stacks) in the case of motion. The image value (brightness) of each pixel inside the pillbox volume is extracted, and the 100 brightest pixels (from each pillbox in each 3D image stack) are averaged to find the raw signal value of the neuron at each time point. This method prevents the signals from depending on the precise placement and size of the user-defined pillboxes, and protects against movement artifacts.

Raw neuron signals are adjusted to show Δ*F*/*F* levels by subtracting the background signal (from a separate pillbox in the larva that is not surrounding any neurons), then dividing by *F*, the minimum signal of the neuron observed during the movie. When comparing across developmental stages and temperature sine wave conditions, we use Δ(Δ*F*/*F*) by taking the difference between the maximum and minimum Δ*F*/*F* values. More precisely we subtract the average of the two minima that occur before and after the peak signal from the peak level, do this for both peaks (since all movies have two periods of sine wave modulation), and average the two values. We note that although we extract signals from individual neurons, in computing average signals under different temperature and developmental stage conditions, we average the signals from all responding neurons together. During immobilization, the orientation and shape of the animal's head are distorted, so it was often difficult to determine the identity of the three cool-sensing neurons in the DOG, especially for older larvae with thicker and more opaque tissue between the objective and the neurons.

### 2.8. Simulated thermal navigation trajectories

Monte Carlo simulations of random walk trajectories were generated in the following way. Each track is given a speed *v*_0_ (equal to the empirical average speed of the developmental stage being simulated). An initial random angle θ_0_ is assigned, then each time step Δ*t* the animal changes position by Δ*x*_*i*_ = *v*_0_ cos θ_*i*_ and Δ*y*_*i*_ = *v*_0_ sin θ_*i*_. After each step, there is a probability of turning *p*_*i*_, which is a function of the crawling direction θ_*i*_ and based on *R*(θ), which for these simulations takes on three discrete values: *R*_*c*_ for crawling in the left quadrant, *R*_*w*_ for crawling in the right quadrant (these are the two input parameters for the simulations), and *R*_⊥_ = (*R*_*c*_ + *R*_*w*_)/2 for crawling in the vertical quadrants.

Random number generation determines (based on *p*_*i*_) whether a turn occurs. If there is a turn, the crawling direction is updated by θ_*i*+1_ = θ_*i*_ ± |Δθ|, where the ± is chosen randomly as +1 or −1 and the turn angle |Δθ| is chosen from a probability distribution based on a histogram of real turn angles measured across all development stages.

Tracks proceed until time elapses (15 min) or the trajectory reaches the edge of the arena, which is 10 cm away from the start point in any cardinal direction.

For each completed track, the navigation index is calculated by dividing the average *x*-velocity 〈*v*_*x*_〉 by the average speed 〈*v*〉. Thousand tracks were generated for each (*R*_*c*_, *R*_*w*_) condition, and for each set the mean and standard deviation of the navigation index were calculated.

## 3. Results

We sought to characterize how the signal response in cool-sensing neurons compares to behavioral response, studying both types of response across the development of the fly larva. We use high-resolution, high-throughput behavior experiments in various thermal environments, along with single-cell-resolution microscopy, to determine sensitivity to temperature and other behavioral features.

### 3.1. Exploration across development mirrors exploration across temperature

**Figure 3:**
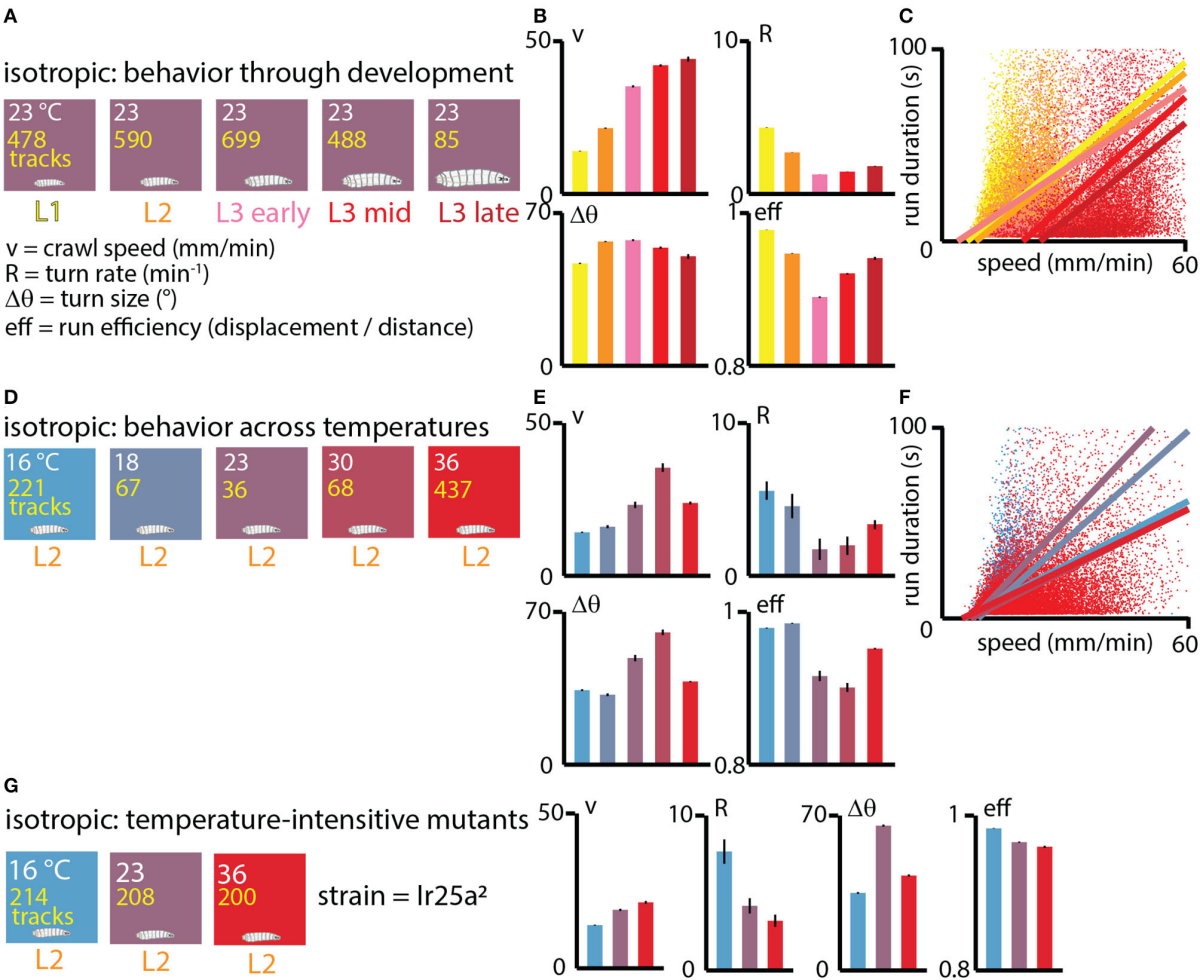
Exploratory behavior across development under isotropic conditions. **(A)** Experiments performed at approximately room temperature (23°C), with wild type (Canton-S) larvae tested throughout development at five ages: first instar (L1, yellow), second instar (L2, orange), early third instar (L3, pink), mid third instar (red), and late third instar (dark red). The number of tracks analyzed is listed in yellow text for each condition. The exploration parameters extracted are the average crawl speed during runs (*v*), the turn rate during runs (*R*), the average size of turns (Δθ), and the average efficiency of individual runs (eff). **(B)** Exploration behavior parameters across development. Average speed (*v*) increases as larvae develop and become larger, while turning rate *R* (or equivalently, the inverse of the average run duration Δ*t*) tends to decrease. **(C)** Scatter plot of run duration vs. crawl speed, for all five developmental stages, where each dot represents an individual run (straight part of a trajectory between two turns), with ~55, 000 runs analyzed. In addition to the overall trend observed in **(B)**, run duration is highly correlated with run speed within individual development stages (linear fits are the solid lines). **(D)** Experiments performed with Canton-S L2 larvae at five spatially uniform temperature conditions. **(E)** Exploration behavior parameters for increasing temperatures. Average speed tends to increase with temperature and turn rate decreases, except at the highest temperature of 36°C. **(F)** Scatter plot of run duration vs. crawl speed for all five temperatures. As with the larva development trend in **(C)**, durations are longer for faster runs, both overall and at each specific temperature condition. Solid lines are linear fits to the scatter plots for each temperature. **(G)** Experiments at three fixed temperatures performed for IR25a^2^ mutant L2 larvae, whose normally cool-sensing DOG neurons lack the proper membrane receptor. Trends in crawl speed (*v*) and turn rate (*R*) vs. temperature are consistent with the temperature dependence in wild-type animals, suggesting that the results in **(E)** are primarily due to the physical effects of temperature, rather than based on sensory response. Error bars (black) represent the standard error of the mean; all bar graphs have error bars, but some are very small.

We first investigated exploratory search behavior in *Drosophila* larvae as they developed, dividing them into five categories based on size and age: L1, L2, early L3, mid L3, and late L3 (see Methods for details). The animals were staged, ~20 at a time, onto flat agar gel in an isotropic, room temperature environment and allowed to crawl for 15 min (Figure 3A). For each condition we calculated the average crawling speed *v*, the turning rate *R*, the average turn size Δθ, and the average run efficiency.

The resulting trends (Figure 3B) show that crawling speed steadily increases as larvae grow and develop, with late stage third instars (L3) having more than 3× the speed of first instars (L1). At the same time, the rate of turning decreases from L1 to early L3 development. Put together (turning less often and moving faster), this causes older larvae to spread out considerably faster from their original location. Trends in turn size are milder, with somewhat larger turns for L2 and L3 larvae compared to L1. Run efficiency (high number indicates a straight path, low number a high curvature path) drops significantly from L1 to L3 stages, but increases during the L3 stage.

The correlation between run duration (inverse of turning rate) and crawling speed, as seen in the scatter plot of Figure 3C, exists across developmental stages, but also within them, as individual fits of Δ*t* vs. *v* for each development level show.

We next investigated what happens to exploratory behavior at different fixed values of temperature. We staged L2 larvae on five different isotropic arenas (16, 18, 23, 30, 36°C) and observed the same exploration parameters (Figure 3D). The general trends largely mirror what happens across development (Figure 3E), where crawling speed increases with temperature and turning rate decreases up to the 30°C condition, while turn size increases and run efficiency decreases. The 36°C condition is the exception, possibly due to approaching the nociceptive regime. As with development, the correlation between run duration and crawl speed is present across all temperatures, and also within the individual isotropic conditions (Figure 3F).

Because we are interested in disentangling the physical effects of temperature from behavior based on sensing temperature, we also tested the exploratory behavior of IR25a mutant larvae under isotropic conditions (Figure 3G). Lacking the receptor IR25a, the cool and warm sensing neurons in the dorsal organ should no longer be temperature sensitive. For L2 mutant larvae, the trends in crawl speed, turning rate, turn size, and run efficiency are very similar to wild type larva, suggesting that modulating exploration parameters could result from the physical effects of temperature in the different isotropic environments.

### 3.2. Thermotaxis strategy effectiveness is maintained until late stage development

**Figure 4:**
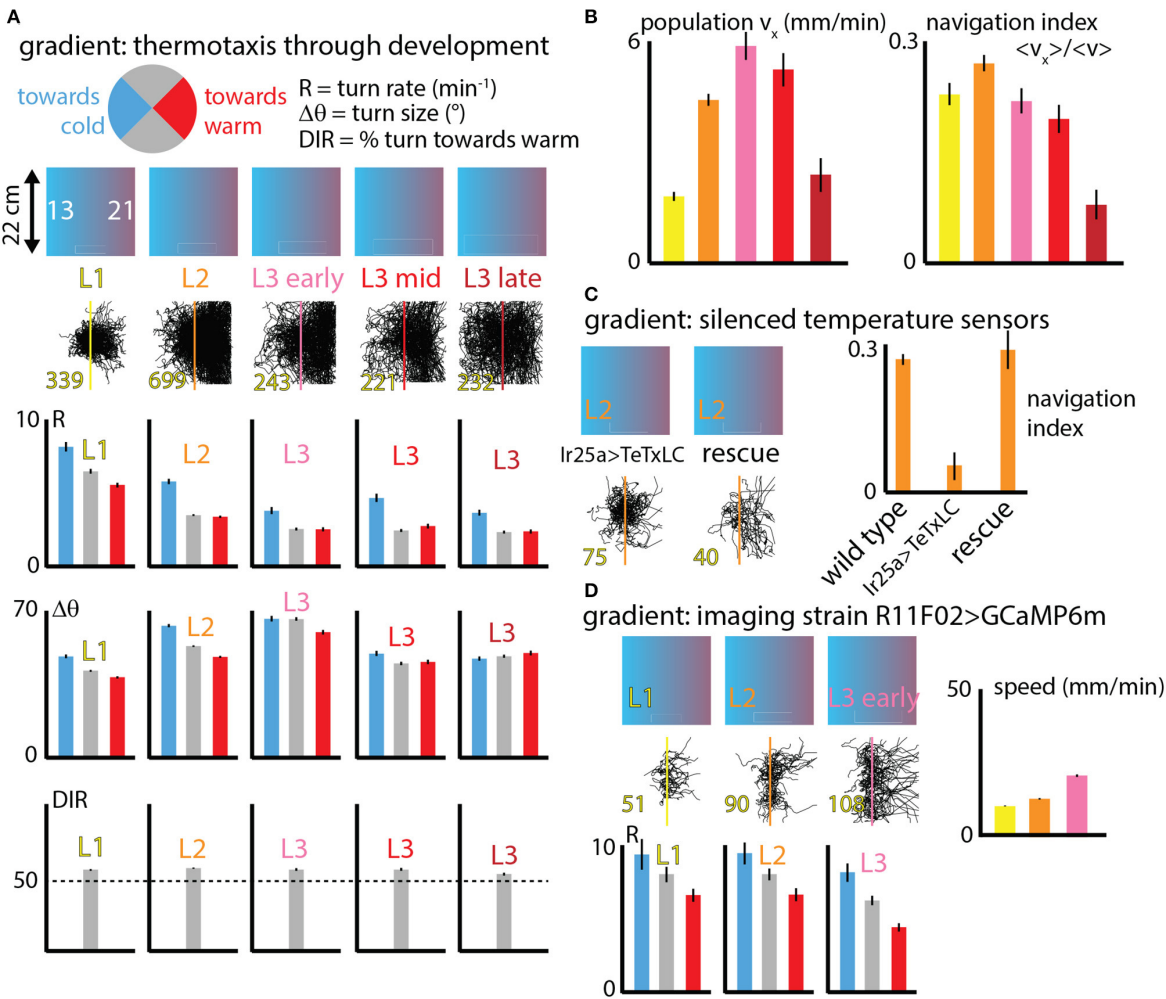
Navigation behavior across development under spatial thermal gradient conditions. **(A)** Three behaviors (turn rate *R*, turn size Δθ, and turn direction) are analyzed for distinct crawling direction ranges (pie chart circle). Larvae at five stages of development (L1, L2, and early, mid, and late L3) are tested on a spatial linear temperature gradient arena that spans from 13 to 21°C across 20 cm (0.4°C/cm). Each column indicates the developmental stage, shows all measured trajectories (black, with the number of tracks in yellow text), and compares *R* and Δθ for larvae crawling toward the warm side of the gradient (red), cold side of the gradient (blue) or perpendicular to the gradient (gray); and the bottom section shows DIR as the percentage of turns that are toward the warm side. **(B)** Navigation summary metrics that result from the behavior component modulation shown in **(A)**. The average *x*-component of crawling velocity (left), i.e., the drift velocity of the population of larvae, increases with developmental stage except for late L3 animals. The navigation index, which is a normalized *v*_*x*_ that isolates the effectiveness of the navigation decisions, is the same for all developmental stages, except late L3 animals. **(C)** Larvae with IR25a-expressing temperature sensing neurons genetically silenced by tetanus toxin were tested on the spatial linear thermal gradient, and show greatly diminished navigation. A rescue strain navigates as well as wild type larvae. **(D)** Larvae used for neurophysiology experiments (expressing GCaMP6m in cool sensing neurons) were also tested on the thermal gradient, for three developmental stages. All three are able to navigate, show similar trends in turn rate (*R*) modulation, and crawl faster as they develop and grow. Error bars (black) represent the standard error of the mean; all bar graphs have error bars, but some are very small.

Going beyond fixed isotropic temperature conditions, we studied how fly larva thermotaxis (specifically cold avoidance) on spatial linear temperature gradients changes across development. Dividing behavior parameters (like turn rate *R*, turn size Δθ and turn direction) into categories based on the crawling direction of individual runs within larva trajectories (Figure 4A), we can delineate how larvae are able to move to the warm side of the gradient by modulating specific components of their behavior.

Larvae turn more frequently when heading to the cold (−*x*) side and less frequently when heading to the warm (+*x*) side, compared to segments of larva trajectories pointing perpendicular to the thermal gradient (±*y* directions). Similarly, larvae deploy larger turns when moving in the −*x* direction, although this effect is diminished in the early L3 stage and disappears for late stage L3. Finally, larvae crawling perpendicular to the thermal gradient are more likely to make turns toward the +*x* direction, an effect that is also diminished in late stage L3 animals.

Figure 4B shows two summary metrics of navigation across development. First the overall average *x*-component of velocity was determined for each stage. This measured *v*_*x*_ is closely equivalent to the drift velocity of the population, i.e., how quickly the center of mass of all larvae moves in the +*x* direction. This *x*-velocity increases dramatically from L1 to L3, and then drops for late stage third instars.

The second metric, the navigation index, is a normalized *v*_*x*_ that isolates the efficiency of navigation decisions. *NI* is a dimensionless quantity that would be equal to +1 if every single larva moved exactly parallel to the gradient in the +*x* direction and never turned (and would be −1 if they all moved in the −*x* direction). Navigation index is zero for either no motion at all or no net horizontal motion (like all the isotropic data in Figure 3). Plotting the navigation index for all development stages we find it to be approximately constant until the late L3 stage. That is, the rules followed by L1 up to mid L3 larvae are not the same (note the *R* graphs in Figure 4A), but they are equally effective given the speeds the animals have at their various sizes. This is explored more precisely in the next section.

We performed two additional sets of control experiments. The first (Figure 4C) measures thermotaxis in L2 larvae with Ir25a-expressing neurons silenced (Ir25a>TeTxLC). Unable to sense warming or cooling in the primary DOG sensory neurons, as expected these larvae navigate extremely weakly, if at all. Both wild type (Canton-S) L2 larvae and L2 larvae with Ir25a rescued navigate strongly. This, along with Figure 3G, suggests that perhaps the temperature-insensitive mutants alter locomotory characteristics due to physical effects of temperature. A second control (Figure 4D) measured thermotaxis across development (up to early L3) in the strain R11F02>GCaMP6m. This strain is used for all our neurophysiology experiments. All three stages show similar turning rate modulations to wild-type strains, with higher turn rate *R* heading toward the cold side of the gradient and lower *R* heading toward the warm side. All three stages can navigate to the warm side, and similar to the wild type larvae characterized in Figure 3B, their speed increases steadily across development.

### 3.3. Simulations of navigation generate a thermotaxis strategy landscape

**Figure 5:**
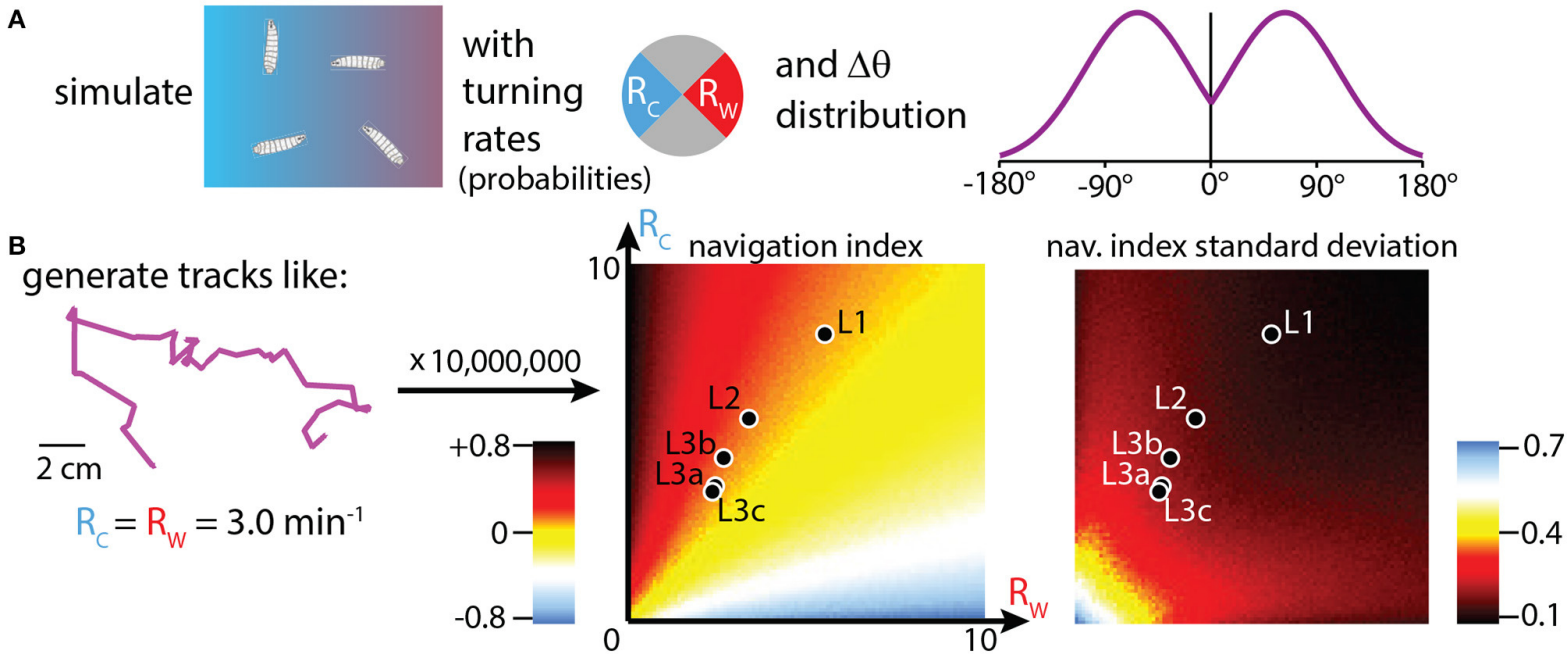
Simulations of thermotaxis: biased random walk trajectories. **(A)** Simulation inputs, where trajectories are built from random walks where the probability of turning during each individual time step depends on the assigned turning rates *R*_*c*_ (toward the cold side of a gradient), and *R*_*w*_ (toward the warm side). The turns are assigned based on the probability histogram shown (Mirrored Gaussian function centered at 63° with a width of 92°). Left or right turns are assigned with equal probability. **(B)** Left: example track built with the simulation methods in **(A)**. Tracks start in the arena center, use constant speeds set to the empirically measured average crawl speeds for each developmental stage, use assigned *R*_*c*_ and *R*_*w*_ values to make turns, and end either after 15 minutes have elapsed or the trajectory encounters the boundary of the 20 × 20 cm arena. We generate 10, 000, 000 trajectories, with 1, 000 tracks simulated for each (*R*_*c*_,*R*_*w*_) condition, incrementing each from 0 to 10 min^−1^ in steps of 0.1. Center: simulation results plotted as a heat map of navigation index for each (*R*_*c*_,*R*_*w*_) combination. The empirical values for each of the five developmental stages (black markers with white outlines) are overlaid on the heat map, showing where each fits in the context of possible navigation results. Right: heat map generated the same way, but showing the standard deviation of navigation index. Animals that turn more have a narrower distribution of outcomes, even if their navigation index is approximately the same.

Adjusting turning rate (*R*) when experiencing various stimulus gradients is how many simple organisms are able to navigate, and it is the primary mechanism for how *Drosophila* larvae accomplish thermotaxis. To isolate this specific behavioral component, and to place the crawling larva behavior in the context of a broader strategy landscape, we generated simulated tracks with over 10, 000 combinations of *R*_*c*_ and *R*_*w*_ (the turn rates during cooling vs. warming). The purpose of these simulations is not to form specific numerical predictions of navigation quality, but to investigate the effectiveness of strategies that modulate *R* across larva development. Simulated tracks are built following the procedure from Figure 5A (also see Methods): each larva moves in a random walk that proceeds in straight lines until a turn randomly occurs (probabilities determined by the chosen *R*_*c*_, *R*_*w*_ values), after which the path randomly turns left or right by an amount drawn from an empirical probability distribution of |Δθ|. After each track ends, its navigation index is calculated. Each *R*_*c*_, *R*_*w*_ condition is simulated 1, 000 times (total over 10, 000, 000 simulated tracks), and the mean and standard deviation of the set of navigation indexes are calculated, then shown as the heat maps seen in Figure 5B.

Real values for *R*_*c*_, *R*_*w*_ are shown within the navigation index and standard deviation heat maps, for each of the five developmental stages. Despite the average turn rates differing by a large fraction across development, with L1 larvae exhibiting very high turn rates for both crawling directions, we see that all five stages are located along the same orange “channel” of the heat map spectrum, a consequence of them all having similar turn rate ratios (*R*_*c*_/*R*_*w*_) of ~1.5. Thus, based on turning rates alone, simulations predict that all five stages would navigate with roughly equally effective strategies. From Figure 4B this holds true except for late stage L3 larvae. The disagreement is likely due to other behavior components; for example, late L3's do not appear to modulate turn size.

The heat map for the standard deviation of navigation index shows differences between developmental stages. The frequent-turning L1 larvae exhibit a much lower range of outcomes than the L3 larvae, with L2 larvae somewhere in between. This can also be seen visually from the trajectories in Figure 4A, where first instar larvae rarely inhabit the left side of the arena even with hundreds of tracks plotted.

### 3.4. Cold sensor response is characterized across development

**Figure 6:**
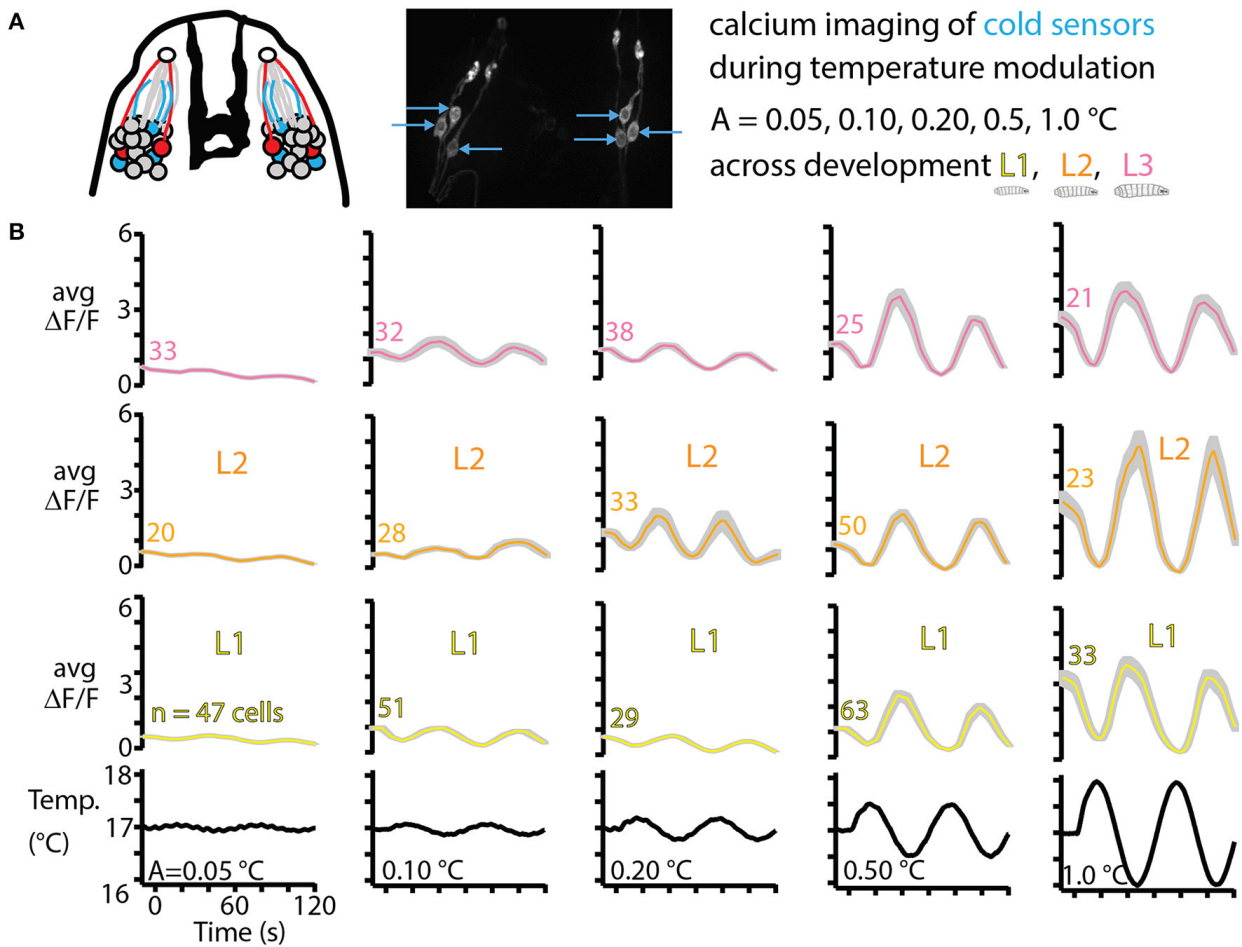
Cold thermosensor response across larval development. **(A)** Left: schematic of the front of the larva's head, featuring the dorsal organ ganglion (DOG) cell body clusters and mouth hooks (black shape in the center). Neurons include olfactory receptor cells (gray), cool sensors (blue) and warm sensors (red), along with their dendrites (colored lines) and the sensilla that interfaces with the outside (white circles). Right: repeat of Figure 2B showing the three cool sensors on each side. These neurons are imaged *in vivo* over time as the temperature of the larva is modulated as sine waves of amplitudes 0.05, 0.10, 0.20, 0.50, and 1.0°C. L1, L2, and L3 larvae are all tested, with ~10 animals for each of the 15 conditions. **(B)** Averaged cool-sensing neuron signals, represented by Δ*F*/*F* vs. time. Numbers next to traces indicate the total number of cells with signals extracted. Gray shading indicates s.e.m. Black traces on the bottom show the temperature of a thermocouple probe underneath the larva's head.

As a counterpart of the thermal navigation experiments in the preceding sections, we also characterized sensory neuron response to temperature changes. Focusing on the primary cooling sensors, three in each DOG in the peripheral nervous system (Figure 6A), we measured neural activity in response to a range of amplitudes of sine wave temperature modulation. The lowest modulation (*A* = 0.05°C, or peak cooling rate −0.0045°C/s) was the smallest sine wave we could generate given the precision of our equipment. The largest modulation (*A* = 1.0°C, or peak cooling rate −0.1°C/s) was safely above cooling rates that would be experienced by larvae crawling on our temperature gradient: an average L3 larva crawling directly in the +*x* direction would feel cooling of −0.03°C/s. We measured neural response for five temperature sine wave inputs, across development for L1, L2, and early L3 larvae.

Average neurophysiology results from 15 combinations of developmental stage and temperature stimulus are shown in Figure 6B. Every condition elicited a measurable response in the neurons of interest, and generally the neuron signals (quantified by Δ*F*/*F*) increased for higher amplitude temperature sine waves, but remained approximately the same across development for any given sine wave.

### 3.5. Larvae are highly sensitive to temperature changes at sensory and behavioral levels

**Figure 7:**
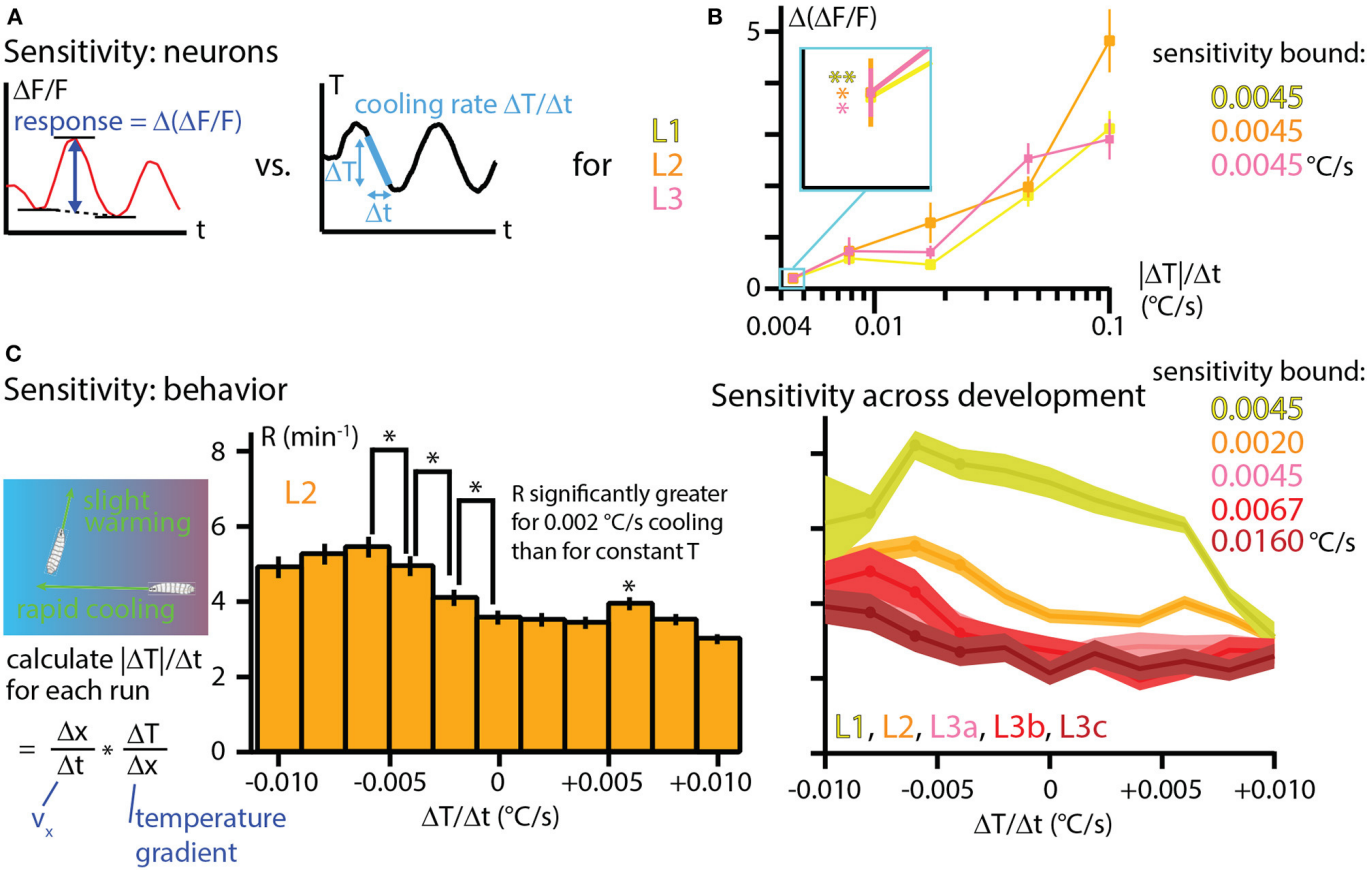
Temperature sensitivity characterized with neurophysiology and behavior across development. **(A)** The response of cool-sensing neurons to temperature modulation is quantified by finding the difference between the average minima in Δ*F*/*F* signal and the peak Δ*F*/*F* signal level. Stimulus strength is quantified as the peak rate of cooling (Δ*T*/Δ*t*) during sine wave modulation. Peak cooling values were extracted from the five temperature sine waves seen in Figure 6. **(B)** Sensitivity curves for L1, L2, and L3 larvae, plotting the average Δ(Δ*F*/*F*) vs. the cooling rate |Δ*T*|/Δ*t*. Neuron response tends to increase for steeper cooling. Inset: expanded view of the average neuron signals at the lowest cooling rate (0.0045°C/s, corresponding to the *A* = 0.05°C temperature sine wave). All three development stages have essentially the same signal levels, and all three are statistically significant signals. *Indicates *p* < 0.05 and ** indicates *p* < 0.01 (Student's *t*-test). **(C)** Sensitivity to temperature change determined by crawling behavior. Left: rates of temperature change (Δ*T*/Δ*t*) are calculated for each individual run during thermotaxis experiments, using the same data shown in Figure 4A. Center: behavioral response to warming or cooling is quantified with the turn rate *R* as a histogram for bins of Δ*T*/Δ*t*. Shown here for L2 larvae. An upper bound on sensitivity is determined by finding that there is a significant difference in turn rate between mild −0.002°C/s cooling and constant temperature (center bar). *Indicates *p* < 0.05 (bootstrapping method, see Methods for details). Right: turning rate histograms for all five developmental stages (a, b, c indicate early, mid, late L3 larvae), shown as line graphs instead of bar graphs. Sensitivity was determined the same way as for L2 larvae, with the upper bounds shown. Error bars and shaded regions indicate s.e.m. for all panels.

An important question in a comprehensive understanding of an animal's response to any stimulus: How sensitive is the response to very small changes in that stimulus? To examine this in our *Drosophila* larva thermal response investigation, we first framed the neurophysiology results from Figure 6 in terms of the peak response to different cooling rates. The difference in fractional signal change between the minimum and maximum values during a sine wave temperature oscillation, Δ(Δ*F*/*F*), was computed for each development stage and amplitude condition (Figure 7A) and then plotted together to form three distinct sensitivity curves (Figure 7B). The general trend of increasing neural signals for increasing temperature changes is apparent for L1, L2, and L3 larvae, with the signals matching closely over the whole range.

Of particular note is the response to the lowest amplitude sine wave, which has peak cooling of −0.0045°C/s (or 0.1°C absolute change). The responses from each of the three developmental stages are indistinguishable, and all of them are significantly above the Δ(Δ*F*/*F*) = 0 level we would expect from a non-responsive neuron. Thus, these measurements place an upper bound in sensitivity of 0.0045°C/s for L1, L2, and early L3 larva stages.

Because larvae crawling on a thermal gradient experience many different warming and cooling rates while exploring and navigating, we sought to determine the whole-animal sensitivity to temperature change by re-examining the thermotaxis data from Figure 4. Splitting the runs from all recorded trajectories into bins of Δ*T*/Δ*t* and computing turning rates, we plot the behavioral response of larvae over a wide range of temperature changes (Figure 7C). In the bar graph example shown, L2 larvae demonstrate significantly different turning rates for the histogram bin centered at 0°C/s compared to the bin centered at −0.002°C/s. We would thus conclude that L2 larvae are sensitive to cooling rates of at least that amount. The trend of a greater turn rate response to greater cooling continues for the next two bins (centered at −0.004 and −0.006°C/s), which seems to establish a consistent effect. We note that the bin centered at +0.006°C/s is also shows a turning rate with a significant difference from adjacent bins. Although it does not appear to be part of a continuing trend, it is possible that this is also a real effect.

We performed a similar analysis for the other larval development stages, finding that mid-L3 and especially late L3 larvae show reduced sensitivity to temperature change, consistent with their weaker navigation index from Figure 4.

We note that in the L2 data from Figure 7C, there is a possible trend where the response starts to diminish as the cooling rate becomes greater (left three bars). Although the adjacent bins do not show statistically significance differences, this could be a legitimate effect. Recent results (Yu et al., 2023) with long time scale larva thermotaxis experiments (up to 6 h) suggest two distinct groups of larvae, one group strongly navigating and the other more neutral. The neutral crawlers would be more likely to crawl in strong-cooling directions (because strong navigators turn away quickly), so perhaps they become more prevalent in the overall averaging for those conditions, leading to a reduced average turning rate.

## 4. Discussion

In this work we have sought to characterize the response of *Drosophila* larvae to changes in temperature, in particular to determine thermal sensitivity at the level of sensory neurons and at the level of overall behavior. Sensitivity has been measured in a similar fashion before (Klein et al., 2015), but with a much smaller data set and only for second instar larvae for behavior and first instar for neural imaging. To our knowledge neither a systematic study of thermotaxis across development with the same experiment type, nor a characterization of sensory neurons across development, has been done before.

Our initial study of baseline behavior for developing larvae under various isotropic conditions yielded two interesting results. First, changes in behavior for developing larvae largely mirror changes in behavior for increasingly warmer larvae at a fixed development stage. This is perhaps not surprising. Older larvae are much, much larger (see photograph in Figure 1A) with the same muscle structure, and thus should crawl faster, and since fly larvae are ectotherms we would expect them to move much faster in warmer environments.

A second interesting result from isotropic experiments is related, and is that the time between turns is strongly correlated with movement speed. Thus, further developed (or warmer) larvae both move faster and turn less often. From a diffusion perspective, speed and run length are the two dominant parameters of a random walk, and these two observed effects work in the same direction and greatly increase the diffusion rate. In an ecological setting this perhaps makes sense, as larger animals take longer to starve and thus it is less risky for them to use energy to explore farther while in search of better conditions. As noted, the turn rate for L1 larvae is very high compared to later development stages. Furthermore, this connection between run duration and speed not only holds across developmental stages (and temperatures) but also within them (Figures 3C, F), suggesting a more universal proprioceptive feedback mechanism that controls turn probabilities. Subsequent studies could examine whether the correlation still holds when using other ways to change crawling speed, such as altering crawling substrate properties.

The follow-up study of directed navigation along thermal gradients (thermotaxis) showed interesting changes related to development. Prior to the late third instar stage, the animals steadily become faster at navigating (as measured by the drift velocity *v*_*x*_), but a more detailed look at the behavioral components and the navigation index metric shows that the efficiency of navigation stays the same across development. That is, L1 and early L3 larvae, for example, are equally effective at directed motion given their crawl speed constraints. This is also evident in the simulation heat map (Figure 5), where all larval stages operate along the same color channel. The overall ability to move to improved thermal conditions thus depends both on the decision-based rules and physical capabilities. We note that because of the run duration vs. speed connection, there is more spread in behavioral outcomes for faster animals.

The random walk style simulations used here allowed us to explore more turning rate parameter space than real experiments and test ideas, but was also a very simple model, using only one type of behavior modulation (turn rate), while ignoring other behavior features. Future studies could take into account modulation of turn size and direction, include drift and curvature within runs (the purple track in Figure 1C is clearly not made of straight lines), or include the time spent during turns. The latter effect would actually further separate diffusion levels between slow and fast crawlers because the slow (higher turn rate) animals would spend more time not crawling. Other past research (such as Luo et al., 2010) has treated simulated crawling by also including turn size and turn direction biases. The other two facets are important, but turn rate modulation had the most prominent effect on navigation outcomes.

We also characterized the primary cool-sensing neurons of the dorsal organ ganglion, measuring calcium activity for a range of temperature change inputs across development. Raw signals were quite dissimilar due to difficulty immobilizing larger larvae and their thicker cuticles, but after normalizing the signal against baseline (Δ*F*/*F*) we found that the sensory neuron responses are all essentially the same across development. At the smallest temperature changes we delivered, the signals from L1, L2, and L3 larvae are nearly identical. The bound on sensitivity was limited by our ability to deliver controlled small temperature changes. This was limited by the precision of the temperature probe and PID controller, and in the future better equipment could determine even lower bounds on thermal sensitivity in these thermosensory neurons.

We were also able to realize small bounds on sensitivity using thermotaxis behavior. By allowing the animals to crawl freely in that arena, they experienced a wide range of temperature changes. A larva crawling slightly off from perpendicular to the thermal gradient will experience very slight, but still quantifiable warming or cooling. Thus, the limitation of sensitivity depends more on the size of the data set and our ability to precisely measure crawling angles, but this is easier than reliably measuring the temperature at the 0.001°C precision level, for example. Future experiments could enhance the measurement capability by using shallower thermal gradients, or a larger space, or slower animals. One could also deploy temporal variation in temperature instead of spatial, with a shallow, steady ramp providing a constant *dT*/*dt* to all crawling animals together.

With both neurophysiology and behavior, we determined extremely high sensitivity to temperature changes, on the order of 0.001°C/s cooling or 0.01°C absolute change. And we should emphasize that the *Drosophila* larva could be much more sensitive, our measurements only provide upper bounds. The exception to sensitivity at the behavioral level is late stage third instar larvae, which experience a decline in several behavioral features that contribute to strong thermotaxis. It is difficult to perform calcium imaging with these larger, stronger (and thicker-cuticled) larvae, but it would be a worthwhile endeavor to attempt to characterize their sensory neurons as well, to see if the sensitivity decline is matched at the cellular level.

The decline in thermotaxis performance in late stage third instar development should also be considered alongside existing literature. There is a general trend where older larvae become less sensitive to external stimuli in general as they focus on moving to pupation sites. Most notable is a paper focused on thermal response (Sokabe et al., 2016), in which the authors investigate thermotaxis in early and late L3 larvae. They find that a “switch in thermal preference” occurs during the L3 stage by observing the final positions of larvae placed on a spatial linear thermal gradient. Early L3 larvae move to warmer conditions and late L3 larvae tend to congregate near 18°C. In our work here, we observe a reduced navigation efficiency in late L3 larvae, but they do still navigate to warmer conditions. Looking more closely at the experimental details of (Sokabe et al., 2016) and our present work, we interpret the findings as consistent with each other. In their experiments, larvae crawl on a steeper linear gradient (1°C/s) centered at 23°C, and the majority of larvae end up in a zone centered at 18°C. In our experiments, larvae start at a colder temperature (17°C) and move relatively slowly, but consistently, toward warmer temperatures. In both cases larvae head toward ~18°C, and our results are complementary, as we use our track analysis to reveal individual behavioral components that produce the overall navigation effect.

Sokabe et al. (2016) also identify rhodopsins (Rh5 and Rh6) in *trpA1* neurons in the central brain that are required for the thermal preference shift to occur. Whether those neurons (noted in Hamada et al., 2008) are directly downstream from the DOG cool-sensing neurons we investigate here is not currently known, but it is possible that they act as separate temperature sensors (as they do in the adult fly, see Gallio et al., 2011). Other work (Luo et al., 2017) determined that TRPA1 is important for sensing temperature change. Taken together, these findings may imply that silencing the DOG cool-sensing neurons does not completely eliminate temperature sensitivity in the larva, so we should not claim definitively that changes in behavior in IR25a mutants (Figures 3, 4) are entirely due to physical effects of temperature.

Overall we hope the work here has identified several important features related to both sensory neuron activity and behavior across development, and can help set the stage for future studies that continue to uncover the details of the stimulus-to-response process in organisms.

## Data availability statement

The raw data supporting the conclusions of this article will be made available by the authors, without undue reservation.

## Author contributions

AE: Conceptualization, Formal analysis, Investigation, Writing—original draft, Writing—review & editing. AF: Data curation, Formal analysis, Investigation, Writing—review & editing. EF: Investigation, Writing—review & editing. JS: Formal analysis, Investigation, Methodology, Writing—review & editing. JF: Formal analysis, Investigation, Writing—review & editing. MK: Conceptualization, Formal analysis, Funding acquisition, Methodology, Project administration, Software, Supervision, Visualization, Writing—original draft, Writing—review & editing.
